# Practical Synthesis of *N*-Formylmethionylated Peptidyl-tRNA Mimics

**DOI:** 10.1021/acschembio.3c00237

**Published:** 2023-07-11

**Authors:** Julia Thaler, Egor A. Syroegin, Kathrin Breuker, Yury S. Polikanov, Ronald Micura

**Affiliations:** † Institute of Organic Chemistry and Center for Molecular Biosciences, University of Innsbruck, Innrain 80-82, 6020 Innsbruck, Austria; ‡ Department of Biological Sciences, University of Illinois at Chicago, Chicago, Illinois 60607, United States; § Department of Pharmaceutical Sciences, University of Illinois at Chicago, Chicago, Illinois 60607, United States; ∥ Center for Biomolecular Sciences, University of Illinois at Chicago, Chicago, Illinois 60607, United States

## Abstract

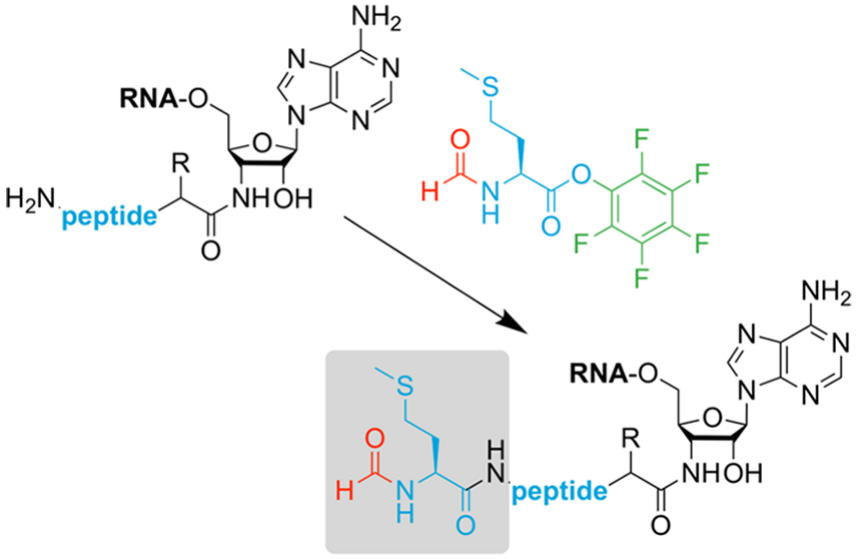

Hydrolysis-resistant RNA-peptide conjugates that mimic peptidyl-tRNAs are frequently needed for structural and functional studies of protein synthesis in the ribosome. Such conjugates are accessible by chemical solid-phase synthesis, allowing for the utmost flexibility of both the peptide and the RNA sequence. Commonly used protection group strategies, however, have severe limitations with respect to generating the characteristic *N*^α^-formylmethionyl terminus because the formyl group of the conjugate synthesized at the solid support is easily cleaved during the final basic deprotection/release step. In this study, we demonstrate a simple solution to the problem by coupling appropriately activated *N*^α^-formyl methionine to the fully deprotected conjugate. The structural integrity of the obtained *N*^α^-formylmethionyl conjugate—and hence the chemoselectivity of the reaction—were verified by Fourier transform ion cyclotron resonance (FT-ICR) mass spectrometry sequence analysis. Additionally, we confirmed the applicability of our procedure for structural studies by obtaining two structures of the ribosome in complex with either fMAI-nh-ACCA or fMFI-nh-ACCA in the P site and ACC-PMN in the A site of the bacterial ribosome at 2.65 and 2.60 Å resolution, respectively. In summary, our approach for hydrolysis-resistant *N*^α^-formylated RNA-peptide conjugates is synthetically straightforward and opens up new avenues to explore ribosomal translation with high-precision substrate mimics.

## Introduction

Protein biosynthesis is a vital cellular process accomplished by molecular machines known as ribosomes.^1−3^ During this process, also known as translation, tRNAs sequentially decode the mRNA harbored in the decoding center of the small ribosomal subunit, whereas their amino acid cargo assembles into a peptide chain in the peptidyl transferase center (PTC). The growing peptide chain exits the ribosome through a long narrow tunnel, which—for a long time—was assumed to only play a passive role in protein synthesis. In recent years, however, evidence arose that the tunnel can take an active role in folding of the nascent peptide chain^4^ and in the regulation of protein synthesis.^5^ For example, macrolide antibiotics bind in the tunnel of the bacterial ribosome and block certain growing peptides, consequently stalling and/or stopping translation.^6−8^ Moreover, many small organic compounds are known to modulate the rate of translation by interacting with the tunnel.^5,9^ Usually, these effects are associated with the nascent peptide of a particular sequence. A few such nascent-chain-arrested ribosome complexes with mRNA, tRNAs, nascent peptide chain, and the respective antibiotic have been structurally characterized. These studies shed the first light on the specific conformations and contacts that are responsible for the effects of the tunnel on translation.^10−15^ Related to this, the ribosomal synthesis of particular peptide sequences is intrinsically difficult, with successive prolines being the most prominent example; the restricted conformational freedom of polyprolines leads to steric interferences, which are considered to be partly responsible for these phenomena.^16^

In previous structural and biochemical studies, the peptidyl-tRNAs of stalled ribosomal complexes were commonly generated *in cis* by exploiting the natural peptidyl transferase activity of the ribosome and its ability to stall in the presence of a drug or a small molecule in the tunnel.^10−13^ This approach generates ribosome nascent chain complexes (RNCs) carrying peptidyl-tRNAs in the P site that contain native (wild-type) chains. What is an advantage on the one hand can be a drawback on the other because these biochemical preparations do not allow for variations in the peptide sequence. Such variations, however, are needed for reasons of comparison, e.g., to the nascent chain in the absence of the antibiotics that triggers stalling or as a consequence of a point mutation in the conserved stalling peptide sequences. Only recently, an important step toward such comparative structure determinations has been reported by capturing nonarrested nascent peptides in the prepeptidyl transfer state using hydrolysis-resistant peptidyl-tRNAs. This became possible by developing a procedure for preparing stable peptidyl-tRNA mimics, which carry an amide instead of naturally occurring ester linkages to prevent spontaneous deacylation of peptidyl-tRNAs during the time course of the experiments. Importantly, such nonhydrolyzable peptidyl-tRNAs are structurally indistinguishable from native tRNA substrates^17^ and are also active in transpeptidation when placed in the A site and combined with native aminoacyl-tRNA in the P site.^18,19^ Moreover, it has been shown that synthetic peptidyl-tRNAs and their short mimics can be efficiently complexed to the ribosome *in vitro* and yet represent a functionally significant state of the PTC.^20,21^ Therefore, amide-linked peptidyl-tRNA mimics represent a reasonable approximation of the reactive state, providing a reliable foundation for the mechanistic hypotheses.

The most convenient large-scale preparation of amide-linked full-length peptidyl-tRNAs follows a recent biochemical protocol^20^ of tRNA^Met^-tailing to replace the 3′-terminal regular adenosine-3′-OH of the CCA end with its amino-substituted adenosine-3′-NH_2_ analogue.^22,23^ Then, the tailed 3′-NH_2_-tRNA^Met^ is enzymatically charged with cysteine by the aminoacyl-tRNA^Met^-synthetase,^22,23^ and finally, native chemical ligation of the thiobenzyl-activated N-formyl-methionyl peptide with cysteinyl-tRNA yields the desired product. The only drawback of this protocol is the restriction of the sequence to cysteine at the C-terminus of the peptide; prolyl-tRNA conjugates are, for instance, not accessible by this approach.

**Figure 1:**
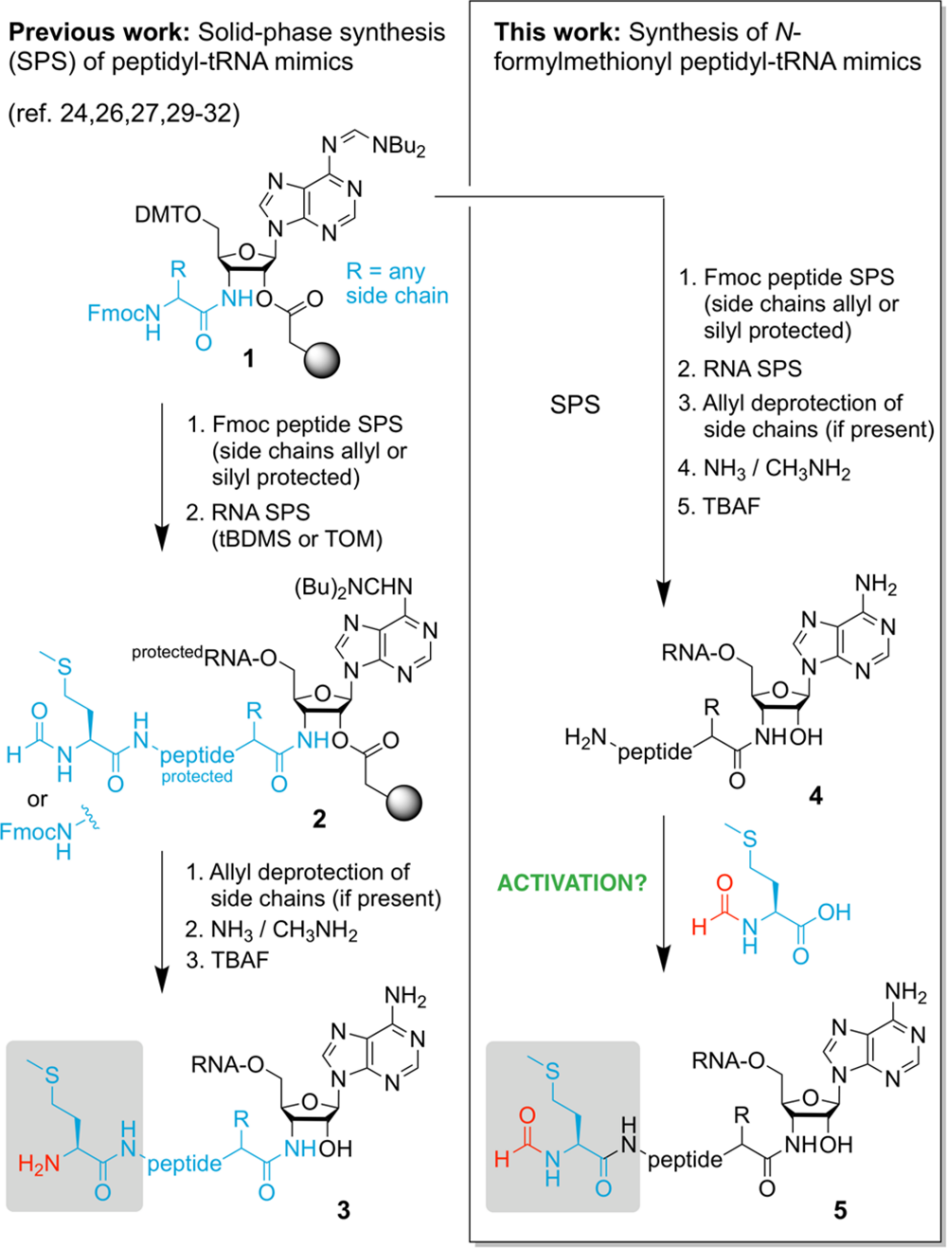
Solid-phase synthesis (SPS) of peptidyl-tRNA mimics with an amide linkage based on support **1**. Previous work has the limitation of lacking the characteristic *N*^α^-formyl group at the *N*-terminal methionine (conjugate **3**) because of deprotection under basic conditions (left panel). This work advances the earlier reported strategy by selective coupling of *N*^α^-formyl methionine to fully deprotected conjugate **4** in solution to obtain target conjugate **5**.

We, therefore, set out to advance a previously developed chemical solid-phase approach that produces complete 3′-amide-linked peptidyl-tRNA fragments **3** (Figure 1, left)^24,25^ that can subsequently be tailored into full-length tRNA with natural tRNA modifications.^26,27^ So far, the limitation of this approach has been the integrity of the characteristic *N*^α^-formyl-methionine terminus because the formyl group of the conjugate assembled on the solid support **1** is typically cleaved during the basic deprotection/release step besides becoming oxidized (S to S=O) during RNA solid phase synthesis when P(III) is transformed into P(V) after each nucleoside coupling (Figure 1, left bottom, steps 2 to 3). Here, we present a simple solution to the problem by attaching an appropriately activated *N*^α^-formyl methionine to fully deprotected conjugate **4** (Figure 1, right panel, steps 4 to 5). This enables efficient synthetic access of previously difficult-to-obtain peptidyl-tRNA mimics which carry the precise N-terminus of a nascent peptide chain, namely, N-formyl methionine.

## Results and Discussion

### Chemical Synthesis of fMet-peptidyl-tRNA Mimics

The synthesis of *N*-formylmethionyl peptidyl-tRNA mimics starts from the previously developed solid support **1** (Figure 1, left panel). This support comprises the adenosine corresponding to the 3′ end A76 of a peptidyl-tRNA and the amide-linked amino acid corresponding to the C terminus of the peptidyl moiety of the same peptidyl-tRNA. The strength of the support is such that any amino acid (natural or modified) can be incorporated. Solid-phase peptide synthesis follows standard *N*^α^-9-fluorenylmethoxycarbonyl (Fmoc) chemistry. Specifically, the side chains of Fmoc amino acid building blocks must be protected either by allyl groups (e.g., allyloxycarbony (alloc) for lysine) or by silyl-labile protecting groups (e.g., *tert*-butyldimethylsilyloxy (TBS) for serine) to be compatible with the protection strategy of RNA solid-phase synthesis that requires acidic conditions for the cleavage of the DMT group in each phosphoramidite coupling cycle. Consequently, typical *tert*-butyl protection or trityl protection of Fmoc amino acid side chains cannot be used. We also note that we preferably apply 2′-O-TBS instead of 2′-O-[(triisopropylsilyl) oxy]methyl (TOM) nucleoside building blocks to avoid any contamination with adducts arising from the reaction with the deprotection byproduct formaldehyde to the primary amino groups of the conjugate that might be encountered if high concentrations of conjugates (**3** or **4**) were used during treatment with TBAF in the final deprotection step.

To obtain target conjugate **5** harboring *N*-formylmethionyl termini, we first considered reagents that had been described in the literature for the formylation of the *N*-terminus of peptides. For instance, ethyl formate, *p*-nitrophenyl formate, and *N*-formylimidazole can be used for this purpose. These reagents are applied against the free *N*^α^H_2_ group (N-terminus), while the side-chain-protected peptide is still attached to the solid support. Then, during amino acid side chain deprotection and release of the peptide from the solid support, which occurs under acidic conditions, the *N*^α^-formyl group remains stable. In contrast, synthetic peptidyl-RNA conjugates are deprotected under basic aqueous conditions and full (or partial) loss of the *N*^α^-formyl group occurs; therefore, we did not further pursue this strategy.

More interesting is the recently introduced reagent of formyloxyacetoxyphenylmethane that has been successfully used for *N*^α^-formylation of free peptides.^28^ However, we anticipated that, in the case of peptide-RNA conjugates, the nucleobase amino groups would also react in such a scenario. Therefore, we opted for an orthogonal strategy that avoids basic conditions and concomitant cleavage of the *N*^α^-formyl group *per se*.

**Figure 2:**
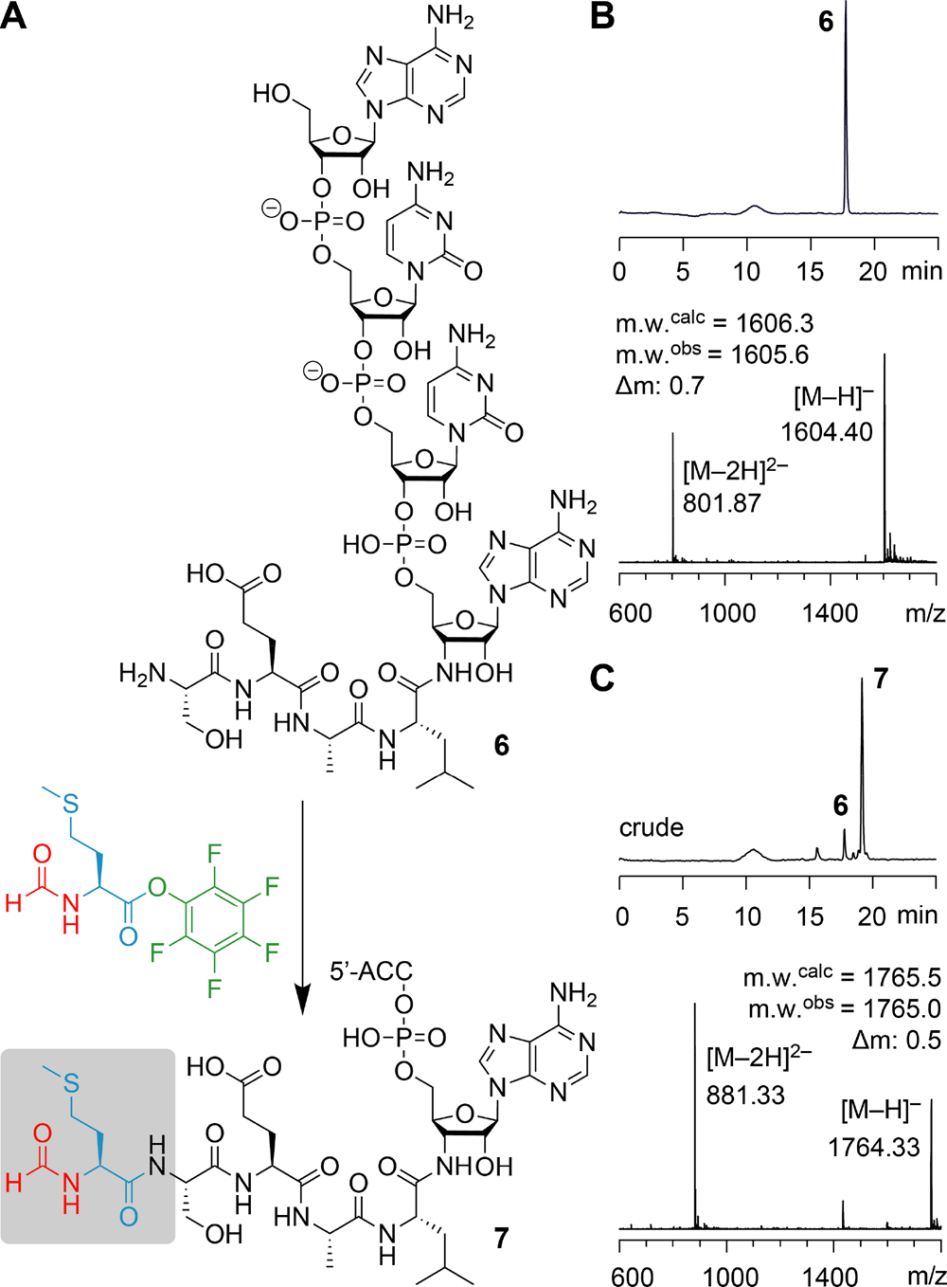
Exemplary *N*-formylmethionylation of a peptidyl-RNA conjugate using *N*-fMet pentafluorophenyl ester. (A) Reaction scheme. (B) Anion exchange HPLC trace and ESI mass spectrum of conjugate **6** (starting material). (C) Anion exchange HPLC trace and ESI mass spectrum of fMet-conjugate (**7**) after conversion using the following conditions: concentration (of **6**) = 100 μM, 20 mM fMet-OPfp, 100 mM Tris·HCl (pH 8) and DMSO (1:1), 37 °C, 15 min, 86%.

The starting point for our undertaking was readily accessible 3′-amide linked peptidyl-RNA conjugates of the general structure **4** (Figure 1).^24,29−31^ We wondered if fMet pentafluorophenyl (Pfp) acid ester is of sufficient reactivity and selectivity to form a peptide bond with the amino group of the N terminus (Figure 2A). Earlier work from our lab already demonstrated that fMet-OPfp can be used to couple fMet directly onto 3′-amino-3′-deoxy modified RNA.^32^ Here, incubation of conjugate **6** with the active ester indeed resulted in a nearly quantitative transformation to a slower migrating species (**7**), as reflected in the anion exchange HPLC traces (Figure 2B and C, Table 1). Isolation of this product and mass spectrometry revealed the expected molecular weight (Figure 2C). Importantly, we had no evidence for side products that could have emerged by reaction with the amino groups of the nucleobases A, C, and G or the 2′-hydroxyl functionalities of the oligoribonucleotide moiety. We therefore continued and prepared the diverse conjugates listed in Table 1 (see also Supporting Figure 1), following the pathway summarized in Figure 1 (right panel) to obtain mimics of the general chemical structure **5**. The peptide sequences of the conjugates were chosen based on our earlier structural investigation of the context-specific action of the classic peptidyl transferase inhibitor chloramphenicol (**12**, **13**),^21^ and, on the other hand, with the goal to contribute to the structural and mechanistic understanding of the protein synthesis with proline residues, which usually impede or even stall translation (**9**–**11**).^33,34^

**Table 1:** Selection of Synthesized Conjugates

#	sequence^a^ peptide (N to C terminus)-NH-3′-RNA-5′	molecular weight calcd [amu]	molecular weight^b^found [amu]	yield^c^ [nmol; %]
**7**	fMSEAL-nh-ACCA	1765.5	1765.2	12 (87)
**8**	fMSEAdL-nh-ACCA^d^	1765.5	1765.0	12 (86)
**9**	fMPP-nh-ACCA	1559.3	1558.5	10 (91)
**10**	fMAPP-nh-ACCA	1630.4	1629.6	11 (97)
**11**	fMAAPP-nh-ACCA	1701.4	1700.6	13 (99)
**12**	fMAI-nh-ACCA	1549.3	1548.6	13 (97)
**13**	fMFI-nh-ACCA	1625.4	1624.5	9 (95)

a For the chemical structures, see Figure 1.

b Determined by ESI mass spectrometry.

c Total amount after purification; percentage (%) of conversion according to HPLC analysis is provided in parentheses.

d Abbreviation dL specifies D configuration of leucine.

### Top-Down MS Analysis of fMet-peptidyl-tRNA Mimics

**Figure 3:**
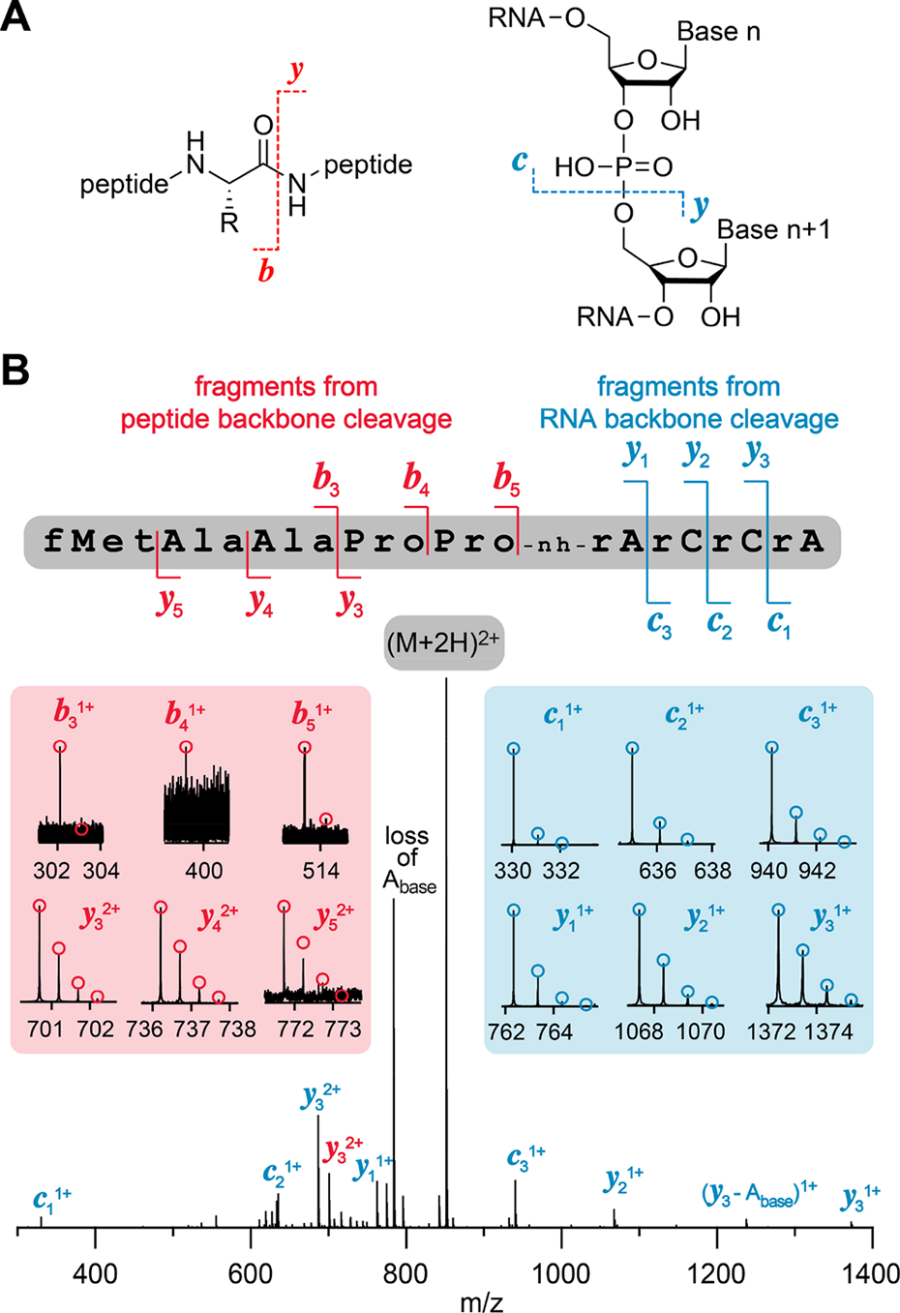
Exemplary ESI FT-ICR mass spectrometric sequence analysis. (A) Collisionally activated dissociation (CAD) of (M + *n*H)^*n*+^ ions of peptides in the collision cell produces *b* and *y* fragment ions from amide backbone bond cleavage, and CAD of both (M – *n*H)^*n*−^ and (M + *n*H)^*n*+^ ions of RNA produces *c* and *y* fragment ions from phosphodiester backbone bond cleavage. (B) Fragment-ion map illustrating sequence coverage from CAD of the peptidyl-RNA conjugate fMAAPP-nh-ACCA **11**. CAD spectrum of the peptidyl-RNA conjugate with the most intensive signal assigned to undissociated (M + 2H)^2+^ ions. The insets show the isotopically resolved fragment signals with assignments of *b* and *y* fragments (cleavage in the peptide moiety) in red and assignments of *c* and *y* fragments (cleavage in the RNA moiety) in blue; calculated isotopic profiles for peptide and RNA fragments are indicated by red and blue open circles, respectively. The complete set of fragment MS signals from CAD of (M + 2H)^2+^ unambiguously confirm the conjugate sequence of fMAAPP-nh-ACCA.

To verify the sequence integrity of the fMet-peptidyl-RNA conjugates, we used electrospray ionization (ESI) Fourier-transform ion cyclotron resonance (FT-ICR) mass spectrometry, which can determine the mass-to-charge ratio (*m*/*z*) of ions with very high precision by measuring the frequency of their cyclotron motion in a static magnetic field. A major strength of this approach is the direct sequencing of biopolymers through backbone cleavage by collisionally activated dissociation (CAD), which produces complementary *b* and *y* fragments for peptides and proteins and complementary *c* and *y* fragments for RNA (Figure 3A).^35,36^ This is typically achieved in the positive ion mode for peptides, while the negative ion mode is used for RNA. Thus, the challenge for top-down sequencing of RNA-peptide conjugates (as provided here) was to find conditions that allow the dissociation of the peptide and RNA backbone in the same experiment. We found that CAD of [M + 2H]^2+^ ions of conjugate **11** provided full sequence coverage in both peptide and RNA moieties (Figure 3B), in agreement with studies of DNA-peptide conjugates,^37^ and the fragment mass values unambiguously confirmed the sequence of fMAAPP-nh-ACCA.

### fMet-peptidyl-tRNA Mimics Bound to Ribosomal PTC

**Figure 4:**
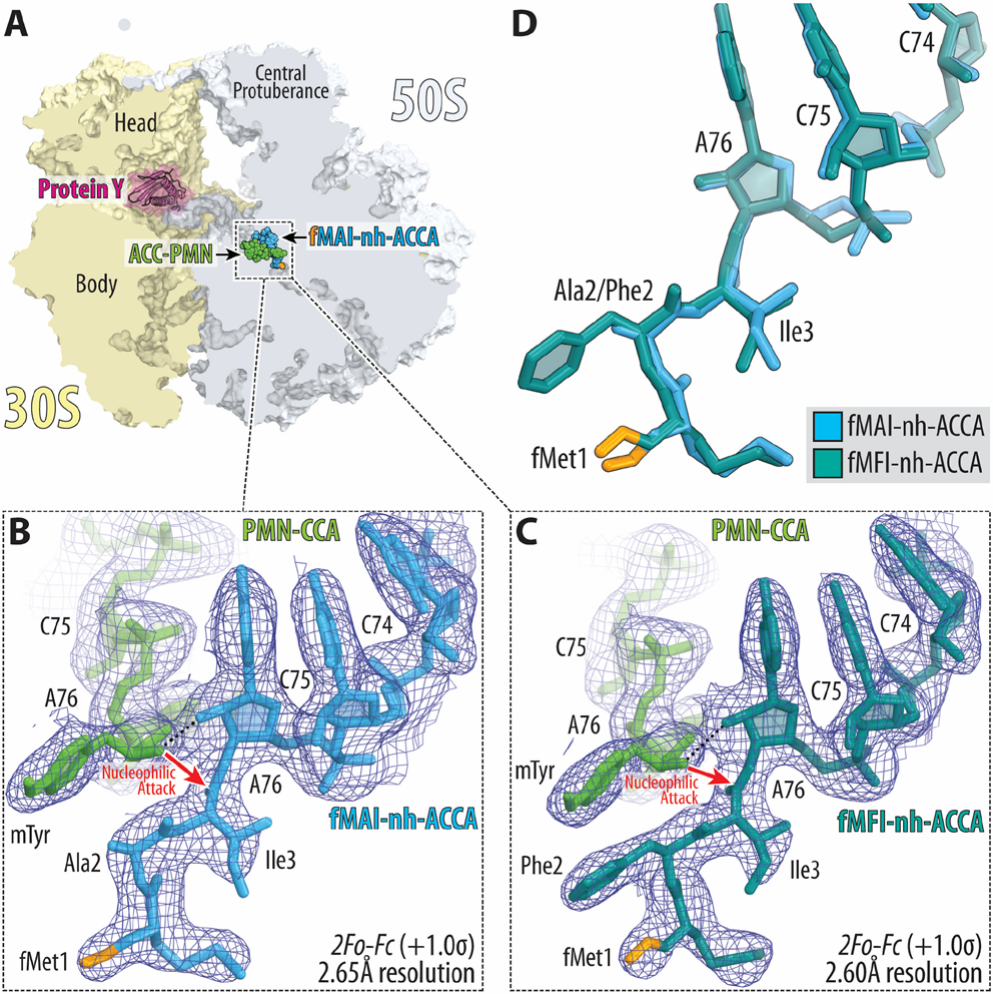
Structures of formylated short tripeptidyl-tRNA analogs in the P site of the 70S ribosome. (A) Overview of the *T. thermophilus* 70S ribosome structures featuring short tRNA analogs viewed as a cross-cut section through the nascent peptide exit tunnel. The 30S subunit is shown in light yellow; the 50S subunit is in light blue. Ribosome-bound protein Y is colored in magenta. (B, C) 2*F*_o_ – *F*_c_ electron difference Fourier maps of PMN-CCA (green) and either formyl-MAI-tripeptidyl-tRNA (B, blue) or formyl-MFI-tripeptidyl-tRNA (C, teal) analogs. The refined models of short tRNA analogs are displayed in their respective electron density maps after the refinement (blue mesh). The overall resolution of the corresponding structures and the contour levels of the depicted electron density maps are shown at the bottom. (D) Superpositioning of the current 70S ribosome structures in complex with short tripeptidyl-tRNA analogs carrying fMAI (blue) and fMFI (teal) tripeptide sequences with each other. Note that the path of the growing polypeptide chain in the exit tunnel is not affected by the nature of the amino acid in the −1 position.

**Figure 5:**
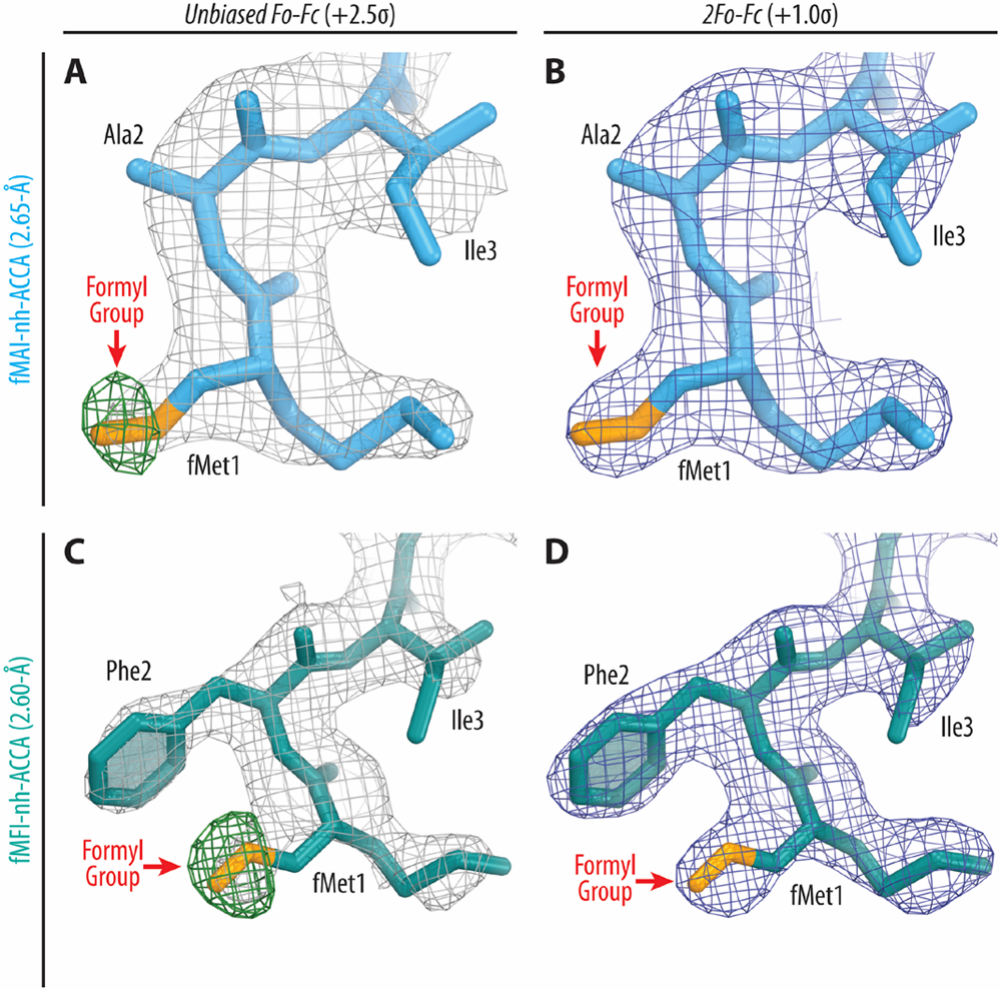
Electron density maps of the ribosome-bound formylated short peptidyl-tRNA analogs. (A, C) Unbiased *F*_o_ – *F*_c_ (gray and green mesh) and (B, D) 2*F*_o_ – *F*_c_ (blue mesh) electron difference Fourier maps of peptide moieties in the *T. thermophilus* 70S ribosome contoured at 2.5σ and 1.0σ, respectively. Gray mesh shows the *F*_o_ – *F*_c_ map after refinement with the entire peptidyl-tRNA analog omitted. Green mesh, reflecting the presence of formyl groups, shows the *F*_o_ – *F*_c_ electron density map after refinement with the nonformylated conjugates. The refined models of fMAI-nh-ACCA (A, B) or fMFI-nh-ACCA (C, D) conjugates are displayed in the corresponding electron density maps.

**Figure 6:**
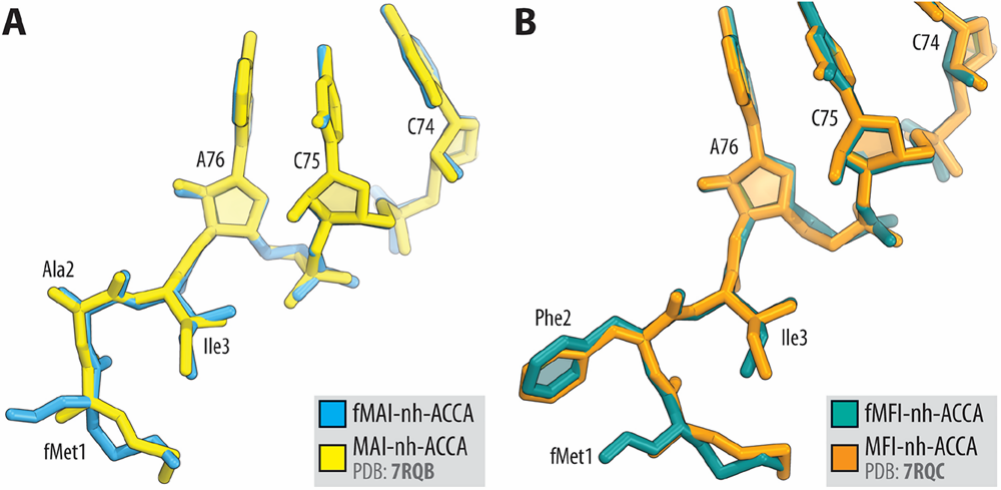
Comparison of the structures of formylated ribosome-bound peptidyl-tRNA analogs fMAI-nh-ACCA (A, blue) or fMFI-nh-ACCA (B, teal) in the P site with the previously reported structures of the same nonformylated conjugates. All structures were aligned based on domain V of the 23S rRNA.

To evaluate if formylation of the N-terminal methionine residue of the peptidyl-tRNA affects the overall conformation of the peptide in the PTC of the bacterial ribosome, we obtained two structures of the 70S ribosome in complex with ACC-Puromycin (PMN-CCA) and formylated fMAI-nh-ACCA (**12**) or fMFI-nh-ACCA (**13**) conjugates as the A- and P-site substrates, respectively (Figure 4). The observed electron density maps for both A- and P-site conjugates in both structures allowed unambiguous modeling of the short aminoacyl- and peptidyl-tRNA analogs (Figure 5). Moreover, the 2.65 Å (for fMAI-nh-ACCA analogue) and 2.60 Å (for fMFI-nh-ACCA analogue) spatial resolution of the obtained maps allowed us to directly visualize formyl groups at the N-termini of the ribosome-bound peptidyl-tRNA mimics (Figure 5A,C). Superpositioning of the new structures containing formylated versions of the peptidyl-tRNA mimics with those without formyl groups obtained before^21^ revealed no significant differences in the overall positions of the peptides (Figure 6).

Our previous studies showed that, in structural terms, these short tripeptidyl-tRNA analogs are indistinguishable from the natural full-length peptidyl-tRNAs in the P site of the ribosome.^20,21^ In particular, the orientation of the attacking α-amino group of the PMN-CCA (aminoacyl-tRNA mimic) relative to the carbonyl carbon of the P-site substrate are identical between the structures harboring full-length tRNAs versus those with short tRNA analogs.^21^ Thus, similar to our previous structures, both new structures represent the prepeptide bond formation state of the PTC (Figure 4B,C), in which peptide moieties of the peptidyl-tRNA analogs adopt similar beta-strand-like conformations, stabilized by the intricate network of H-bonds between their main-chain atoms and the universally conserved nucleotides U2506, G2061, and A2062 of the 23S rRNA.^20,21^ Taken together, our data suggest that the presence of the *N*-formyl group on tripeptidyl-tRNA mimics does not affect the position of the peptide in the ribosomal exit tunnel.

## Conclusions

We have developed a straightforward approach to produce hydrolysis-resistant RNA-peptide conjugates that are *N*^α^-formylmethionylated. Such conjugates mimic naturally occurring peptidyl-tRNAs and are needed for structural and functional studies of ribosomal translation to provide mechanistic insight into protein synthesis. One limitation in generating such conjugates by standard chemical solid-phase synthesis (SPS) has been the incompatibility of SPS with the introduction of a formyl group at the N-terminus of the peptide moiety. In this work, we overcome this challenge by finding the appropriate activation of fMet (in the form of its Pfp ester) to cleanly couple onto a free (unprotected) peptidyl-RNA conjugate. This transformation is equivalent to the direct formylation of a peptidyl-RNA conjugate precursor that would already contain methionine at the N-terminus; however, it has the advantage of a higher molecular weight increase, which makes HPLC product analysis straightforward because of more significant differences in retention times. Even more advantageous is that the presented approach masters the problem of methionine oxidation (thioether to sulfoxide; see, e.g., Figure 3 in reference (24)) that usually occurs during SPS of such conjugates because of repeated exposure to I_2_ solutions required for PIII-to-PV oxidation. In the new approach, the N-terminal methionine is coupled *after* solid-phase synthesis and hence is not exposed to oxidative reaction conditions. The method is particularly valuable because the synthetic precursor conjugates are broadly accessible, with hardly any sequence restrictions. The only limitation concerns lysines that contain a primary amino group at their side chains and that are formylmethionylated without a proper protection strategy. Conceptually, lysine-containing conjugates are accessible by this approach only if a photolabile protection group is applied at the N(ε) position that becomes finally cleaved after fMet has been coupled to the N peptide terminus.

In summary, our path toward hydrolysis-resistant *N*^α^-formylated RNA-peptide conjugates opens up new avenues to explore protein synthesis with high-precision substrate mimics. Although the present showcase of fMFI and fMAI conjugates indicates that the formylation does not alter the binding mode and conformation of these particular peptides, and thus, the biological impact of these findings is relatively modest, it is important that this has been assessed directly, and also, this may not be the case for other peptides.

## Methods

### Preparation of Peptidyl-RNA Precursors **4** with NH_2_ Termini

All peptidyl-RNA conjugates of type **4** were produced and purified following references (24), (29), and (30). The assembly of the conjugates was based on Fmoc peptide solid-phase synthesis and RNA solid-phase synthesis using 2′-*O*-[(triisopropylsilyl) oxy]methyl (TOM)- or 2′-*O*-[*tert*-butyldimetylsilyl (TBDMS)-protected nucleoside building blocks.

*Additional Remark on the Deprotection of Peptidyl-RNAs Containing Allyl Protected Glutamic Acid*. Allyl deprotection was performed in analogy to reference (29). After conjugate assembly, the solid support was treated in the synthesis cartridge with a solution of *N*-methyl morpholine (37 μL, 0.34 mmol) and acetic acid (37 μL, 65 mmol) in chloroform (amylene stabilized; 1 mL). After the addition of tetrakis(triphenylphosphine) palladium(0) (12 mg, 0.01 mmol), the suspension was agitated for 5 h at RT. Subsequently, the solid support was washed with chloroform (3 × 2 mL), dried under a vacuum, and subjected to standard acyl and 2′-*O*-silyl deprotection; see references (24), (29), and (30).

### Synthesis of fMet Peptidyl-tRNAs **5** (i.e., Conjugates **7** to **13**)

*N*-Formyl-l-methionine pentafluorophenylester was synthesized as described in references (32). One equivalent of 3′-amino-3′-deoxyoligoribonucleotides **4** (final concentration = 0.1 mM) and 200 equiv of *N*-formyl-l-methionine pentafluorophenylester (final concentration = 20 mM) were dissolved in 100 mM Tris–HCl (pH 8.0) and dimethyl sulfoxide (1/1, v/v). The typical total reaction volume amounted to 150 μL. After 15 min at 37 °C, the reaction mixture was diluted with 450 μL water and directly applied on a size-exclusion chromatography column (GE Healthcare, HiPrep 26/10 Desalting, 2.6 × 10 cm, Sephadex G25). By elution with H_2_O, the conjugate-containing fractions were collected and evaporated to dryness, and the residue was dissolved in H_2_O (1 mL). Analysis of the crude products was performed by anion-exchange chromatography on a Dionex DNAPac PA-100 column (4 × 250 mm) at 60 °C (flow rate: 1 mL min^–1^; eluent A, 25 mM Tris-HCl (pH 8.0) and 20 mM NaClO_4_ in 20% aqueous acetonitrile; eluent B, 25 mM Tris-HCl (pH 8.0) and 0.60 M NaClO_4_ in 20% aqueous acetonitrile; gradient: 0–35% B in A within 30 min; UV detection at λ = 260 nm).

### Purification of fMet peptidyl-tRNAs **5**

If the conversion reaction yielded less than 80% of the fMet peptidyl-RNA, the conjugates were additionally purified on a semipreparative Dionex DNAPac PA-100 column (9 × 250 mm) at 60 °C with a flow rate of 2 mL min^–1^ (for eluents, see the section Synthesis of fMet Peptidyl-tRNAs **5**). Fractions containing the conjugate were concentrated to near dryness and diluted with 0.1 M (Et_3_NH)^+^HCO_3_^–^ and loaded on a C18 SepPak Plus cartridge (Waters, Millipore), washed with H_2_O, and eluted with H_2_O/CH_3_CN (1:1). Conjugate-containing fractions were evaporated to dryness and dissolved in H_2_O (1 mL). The quality of the purified conjugate was analyzed by analytical anion-exchange chromatography (for conditions, see the section Synthesis of fMet Peptidyl-tRNAs **5**). The molecular weight of the synthesized conjugate was confirmed by liquid chromatography-electron ionization (LC-ESI) mass spectrometry (Table 1). Yields were determined by UV photometrical analysis of conjugate solutions.

### FT-ICR Mass Spectrometry Analysis of fMet Peptidyl-RNA Products

Experiments were performed on a 7T Fourier transform ion cyclotron resonance (FT-ICR) mass spectrometer (Bruker APEX ultra) equipped with an ESI source and a collision cell through which a flow of Ar gas was maintained for CAD. Peptide-RNA conjugates were electrosprayed (flow rate 1.5 μL/min) from 1 μM solutions in 1:1 H_2_O/CH_3_OH vol/vol with 1% acetic acid as an additive. Methanol (Acros) and acetic acid (Fisher Scientific) were HPLC grade, and H_2_O was purified to 18 MΩ·cm at RT using a Milli-Q system (Millipore). RNA concentration was determined by UV absorption at 260 nm using a NanoPhotometer (Implen). Prior to dissociation by CAD, the (M + *n*H)^*n*+^ ions under study were isolated in a linear quadrupole; for a more detailed description of the experimental setup for CAD, see reference (38).

### Crystallographic Structure Determination

X-ray crystal structures of 70S ribosomes from *Thermus thermophilus* in complex with protein Y and short tRNA mimics were determined as described previously, see reference (21). The statistics of data collection and refinement are compiled in Supporting Information Table 1. All figures showing atomic models were rendered using PyMol software (www.pymol.org).

## Data Availability

Coordinates and structure factors were deposited in the RCSB Protein Data Bank with accession codes 8T8B for the *T. thermophilus* 70S ribosome in complex with protein Y, A-site aminoacyl-tRNA analog ACC-Pmn, and P-site peptidyl-tRNA analog fMAI-nh-ACCA; 8T8C for the *T. thermophilus* 70S ribosome in complex with protein Y, A-site aminoacyl-tRNA analog ACC-Pmn, and P-site peptidyl-tRNA analog fMAI-nh-ACCA;

## Supporting Information Available

Statistics of crystallographic data collection/refinement and further characterization data of conjugates (PDF)
