# Supplementary material for: Practical Synthesis of N-Formylmethionylated Peptidyl-tRNA Mimics
Source: ACS Chem Biol. 2023 Jul 11;18(10):2233–9. doi: 10.1021/acschembio.3c00237 (PMC10594587; doi:10.1021/acschembio.3c00237)
Supplement: Supplementary file 1 — cb3c00237_si_001.pdf [file cb3c00237_si_001.pdf]

Supplementary Information for

Practical synthesis of *N*-formylmethionylated peptidyl-tRNA mimics

Julia Thaler,[†,⊥] Egor A. Syroegin,[§,⊥] Kathrin Breuker,[†] Yury S. Polikanov,[§,$,+,*] and Ronald Micura[†,*]

[†] Institute of Organic Chemistry and Center for Molecular Biosciences, University of Innsbruck, Innrain 80-82, 6020 Innsbruck, Austria.

[§] Department of Biological Sciences, University of Illinois at Chicago, Chicago, IL, USA.

[$] Department of Pharmaceutical Sciences, University of Illinois at Chicago, Chicago, IL, USA.

[+] Center for Biomolecular Sciences, University of Illinois at Chicago, Chicago, IL, USA

* ronald.micura@uibk.ac.at, * yuryp@uic.edu

Contents

Supporting Figures

Figure S1 S2

Supporting Tables

Table S1 S3

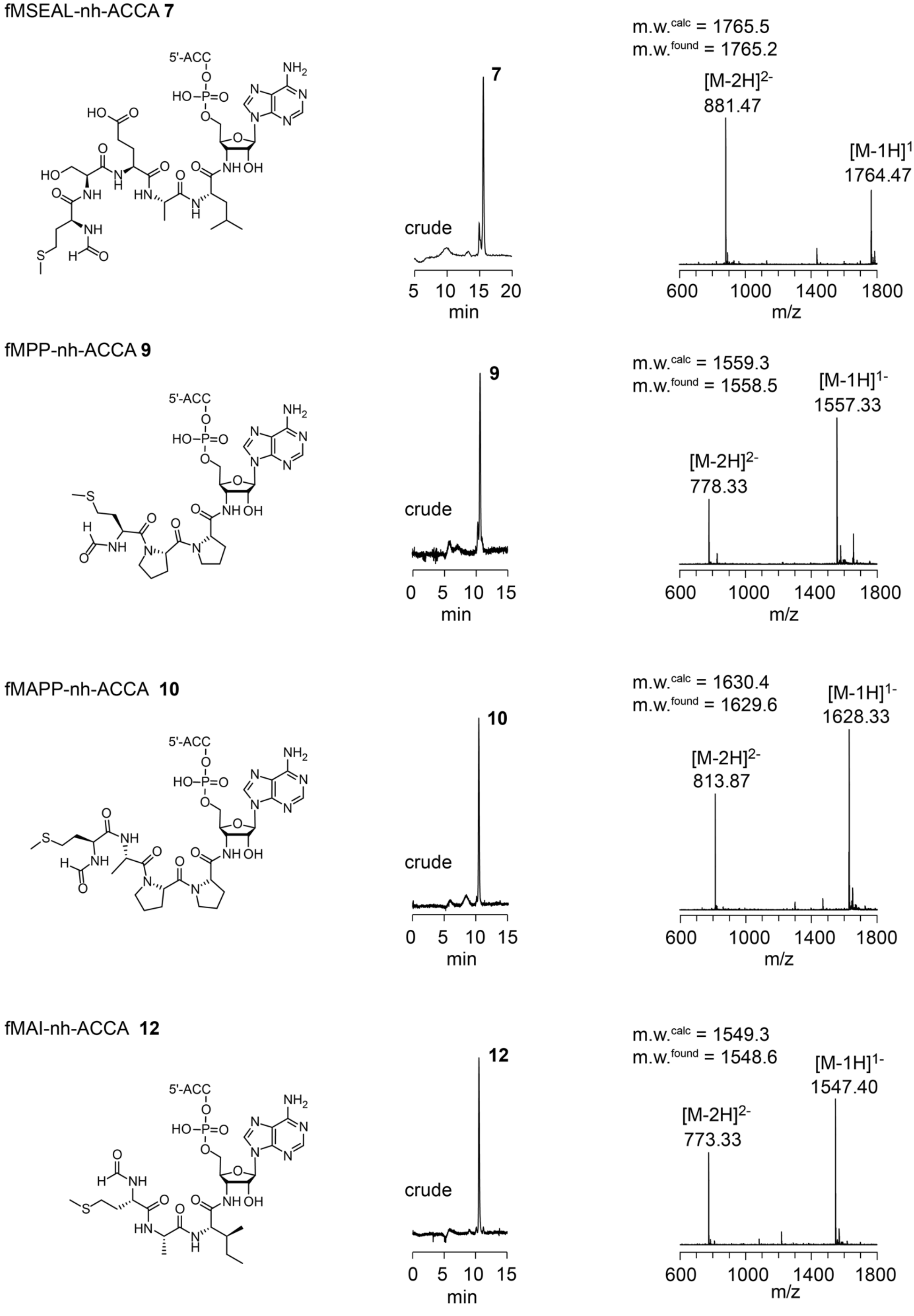

Figure S1. Examples for *N*-formylmethionylation of peptidyl-RNA conjugates using *N*-fMet pentafluorophenyl ester; chemical product structures (right); anion exchange HPLC traces of crude reaction mixtures (middle); ESI mass spectra of conjugates (right). Conditions of conversion: 100 µM conjugate, 20 mM fMet-OPfp, 100 mM Tris·HCl (pH 8) and DMSO (1:1), 37 °C, 15 min.

Table S1. X-ray data collection and refinement statistics.

Crystals		70S-PY complex with PMN-CCA and fMAI-nh-ACCA<br>PDB entry 8T8B	70S-PY complex with PMN-CCA and fMFI-nh-ACCA<br>PDB entry 8T8C
Diffraction data			
Space Group		$P2_12_12_1$	$P2_12_12_1$
Unit Cell Dimensions, Å (a x b x c)		207.11 x 441.13 x 612.49	209.83 x 450.39 x 621.88
Wavelength, Å		0.9795	0.9795
Resolution range (outer shell), Å		358-2.65 (2.72-2.65)	162-2.60 (2.67-2.60)
I/σI (outer shell)		8.02 (1.02)	8.84 (1.01)
Resolution at which I/σI=1, Å		2.65	2.60
Resolution at which I/σI=2, Å		2.85	2.83
CC(1/2) at which I/σI=1, %		16.9	18.0
CC(1/2) at which I/σI=2, %		45.0	45.0
Completeness (outer shell), %		99.0 (99.1)	99.5 (99.5)
R_{merge} (outer shell)%		19.0 (193.9)	15.4 (128.7)
No. of crystals used		1	1
No. of reflections	Observed	9,752,234	8,726,049
	Unique	1,585,401	1,770,773
Redundancy (outer shell)		6.15 (5.72)	4.93 (4.46)
Refinement			
Resolution range of the diffraction data included in the refinement, Å		119-2.65	122-2.60
No. of reflections used in the refinement		1,584,840	1,770,705
R_{work}/R_{free}, %		21.1/25.7	22.0/26.9
No. of Non-Hydrogen Atoms			
RNA		193,589	193,597
Protein		93,169	93,181
Ions (Mg, K, Zn, Fe)		2,456	2,518
Waters		5,789	7,080
Ramachandran Plot			
Favored regions, %		91.90	91.65
Allowed regions, %		7.19	7.58
Outliers, %		0.91	0.77
Deviations from ideal values (RMSD)			
Bond, Å		0.004	0.004
Angle, degrees		0.845	0.849
Chirality		0.042	0.043
Planarity		0.005	0.005
Dihedral, degrees		16.751	16.561
Average B-factor (overall), $Å^2$		73.0	57.5
